# A Novel Somatic Treatment for Post-traumatic Stress Disorder: A Case Report of Hydrodissection of the Cervical Plexus Using 5% Dextrose

**DOI:** 10.7759/cureus.23909

**Published:** 2022-04-07

**Authors:** K. Dean Reeves, Jessica Shaw, Rebekah McAdam, King Hei Stanley Lam, Sean W Mulvaney, David Rabago

**Affiliations:** 1 Physical Medicine and Rehabilitation, Private Practice, Roeland Park, USA; 2 Medical School, University of Kansas School of Medicine, Kansas City, USA; 3 Medical School, University of Kansas Medical School, Kansas City, USA; 4 Musculoskeletal Medicine, The Hong Kong Institute of Musculoskeletal Medicine, Kowloon, HKG; 5 Family Medicine, The Chinese University of Hong Kong, New Territories, HKG; 6 Medicine, Uniformed Services University, Bethesda, USA; 7 Family and Community Medicine, Penn State College of Medicine, Hershey, USA

**Keywords:** veterans, complex-ptsd, ptsd diagnosis and treatment, dextrose hydrodisssection, post traumatic stress disorder (ptsd)

## Abstract

Despite years of standard treatments, a Marine veteran and former firefighter, disabled due to severe post-traumatic stress disorder (PTSD), worsened over ten weeks while receiving usual care. Bilateral injection of 10 mL of 5% dextrose in water using a 30-gauge needle just under the sternocleidomastoid muscle was performed at weeks 10, 12, 14, 16, and 18. Clinically important improvements were observed by 18 weeks on a 0-10 anxiety rating scale (57%), the PTSD checklist for civilians (41%), EuroQol overall quality of life scale (40%), and the Hospital Anxiety and Depression Scale (28%). Improvements were stable through 22 weeks. He reported symptomatic benefit on anxiety within 20 minutes of injection, suggesting a neurogenic mechanism, potentially related to a therapeutic effect on the nearby sympathetic trunk/superior sympathetic ganglion. Advantages of this procedure over stellate ganglion blockade include its safety (no lidocaine), bilateral treatment option, simplicity, and comfort.

## Introduction

Post-traumatic stress disorder (PTSD) is a well-recognized debilitating mental health condition associated with prior exposure to trauma [1]. The international prevalence of moderate PTSD in health care workers exceeds 21%, with the COVID-19 pandemic a likely contributing factor [2]. For example, a recent survey (n = 4,022) of emergency medical services personnel revealed that 6.6% had attempted suicide and 37% experienced thoughts of suicide [3]. The prevalence of PTSD is likely underreported. Many do not seek treatment due to concerns with the stigma of PTSD and fear of job loss [4]. The number of people living with PTSD who do not seek psychotherapy or receive insufficient treatment approaches 50% [5]. Recent evidence suggests that multimodal care is more effective in treating PTSD symptoms than a single therapy approach [6]. Combining treatments with complementary mechanisms of action may optimize patient outcomes [7].

Perineural hydrodissection involves the injection of fluid placement under high-resolution ultrasound guidance about symptomatic peripheral nerves, with a focus on separating the nerves from areas of fascial or other connective tissue compression/entrapment [8]. This is most commonly performed using dextrose 5% in water (D5W), as, in addition to a mechanical effect, it is thought to have an ameliorative effect on neurogenic inflammation (upregulation) of C fibers, such as those found in the cervical sympathetic ganglia [9].

Lam et al. included patients with a concurrent diagnosis of PTSD in a case collection of outcomes from D5W perineural hydrodissection for chronic pain, and anecdotally observed that several patients expressed improvement in their PTSD symptoms [10]. Although effects of treatment on the level of PTSD symptoms were not measured in that study, the lead author of this case report observed that chronic pain patients with a concurrent diagnosis of PTSD spontaneously described improvement in noise intolerance, light intolerance, and anxiety within minutes of injection. Using the PTSD checklist for civilians (PCL-C) for screening before dextrose hydrodissection of the cervical plexus (DHD-CP), three consecutive patients with a PCL-C > 50 (mean 60±8.8; range 54 to 70 points) rated their anxiety improvement 20 minutes post-treatment as 4.6 on a 10-point numerical rating scale (8.3 to 3.7; a 55% reduction). Our clinical experience was provocative and suggested a potential therapeutic benefit from DHD-CP. Although PTSD and chronic pain often co-occur [11], the next patient we saw became aware of potential treatment in our clinic, did not have chronic pain, and we gathered his data more meticulously and specifically for presentation as a case report of effects in a patient absent potential confounding effects of chronic pain.

## Case presentation

This 53-year-old male patient had served in the Marine Corps for six years and subsequently worked as a paramedic/firefighter. Chronic pain was not an issue for him, rated at 1/10. Unfortunately, he retired as a firefighter due to intractable PTSD symptoms, which included emotional disruption, dissociation, insomnia with nightmares, and inability to concentrate. Because of these profound effects on activities of daily living, he was in the process of applying for social security disability at the time of his first visit. He denied recent suicidal ideation, but personal despair was marked. The patient's psychiatrist confirmed no indication of a formal psychiatric disorder other than PTSD, although secondary anxiety and depression were present.

Medication history included sertraline, paroxetine, prazosin, alprazolam, bupropion, desvenlafaxine, and aripiprazole. At presentation, he was taking trazodone, fluoxetine, and quetiapine. Non-medication treatments received included cognitive processing therapy, cognitive behavioral therapy, prolonged exposure therapy, eye movement desensitization and reprocessing, meditation, group therapy, and hospitalization for 39 consecutive days. He had completed five months of prolonged exposure treatment and cognitive processing therapy. 

We first administered a Traumatic Life Events Questionnaire [12] to reveal the breadth of traumatic exposure and to identify the single event that most consistently intruded on the patient’s consciousness. Consistent with current evidence-based guidelines for the diagnosis of PTSD, we then used that event for the completion of the Clinician-Administered PTSD Scale (CAPS) [13]. The CAPS has been extensively validated, is a widely used and structured diagnostic interview approach [13], and corresponds with the latest edition of the Diagnostic and Statistical Manual of Mental Disorders (DSM-5) [14]. The patient's life experience revealed a broad range of traumatic events with direct or indirect contact with 13 of 16 categories of trauma. The CAPS score was 61, corresponding to the severe range of PTSD [13].

Current anxiety, measured on a 0-10 numerical rating scale (NRS), was 7/10. The PCL-C is a valid and reliable measure of PTSD symptoms with good consistency and reliability [15] and is commonly used to measure PTSD treatment response [16]. The PCL-C score for this patient was 73 points out of a possible 85. The Hospital Anxiety and Depression Scale (HADS) is a scale with good consistency and reliability [17] used to measure psychological distress, with a threshold of 12 points for both anxiety and depression subscales indicative of "substantial distress" [18]. The patient's scores were 18 and 14 for anxiety and depression, respectively. The EuroQol 0-100 overall quality of life score was 50, consistent with a moderately-to-severely-impacted quality of life. The EuroQol 5-dimension, 5-level, plus cognition scores revealed moderate to extreme effects in four of six dimensions: anxiety-depression, concentration-memory, usual activities, and pain-discomfort [19].

Two weeks prior to presentation to this clinic, this patient’s psychiatrist increased his fluoxetine and quetiapine dosages from 40 to 60 mg, and 100 to 150 mg respectively. The effects of a dosage increase of sertraline on symptoms of anxiety or depression, and of quetiapine as an adjunctive treatment for PTSD on CAPS score and hyperalertness state, reach their maximum effect by 10 [20, 21] and eight weeks [22], respectively. Therefore, we delayed an additional 10 weeks prior to initiating injection treatment to observe for any potential effect of these medication changes in his baseline status. 

At weeks 10, 12, 14, 16, and 18 after presentation, 10 mL of D5W, diluted with sterile water (Hospira, Lake Forest, USA; NDC 00409-4887-10) from 50% dextrose (Hospira; NDC 0409-6648-16), was placed just deep (dorsal to) the sternocleidomastoid (SCM) muscle and lateral to the carotid sheath under ultrasound guidance, using a 30-gauge 2.5 cm needle, at the level of the cervical plexus, which can be approximated by the level of the mandible. Needle entry is close to the posterior edge of the SCM rather than behind it to avoid the cervical plexus exit point, and directed almost vertically (60-90 degrees pointing anteriorly) at an angle steep enough to allow the short needle to reach the necessary depth depending on neck size. Hydrodissection with needle advancement is recommended for comfort and safety. Optimally, needle entry should be adjusted superiorly or inferiorly to miss the visualizable external jugular vein and greater auricular nerve, both of which pass over the SCM. This will avoid leaving a bruise or causing a temporary unpleasant sensation about the ear.

**Figure 1:**
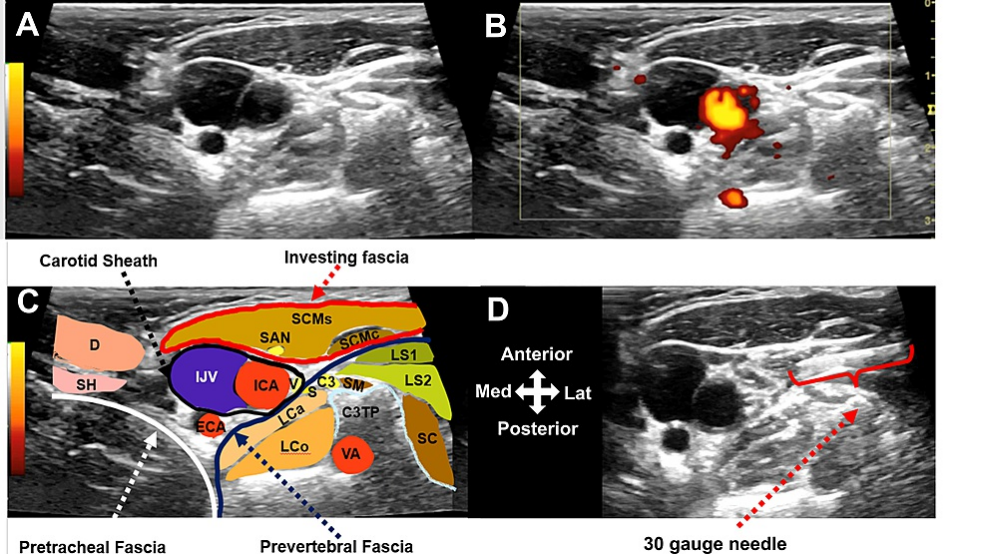
High-resolution ultrasound over the sternocleidomastoid at C3 level on the right. A: Standard B mode ultrasound image B. Doppler mode C. Labeled image: C3, C3 nerve root; C3TP, transverse process of C3; D, digastric muscle; ECA, external carotid artery; ICA, internal carotid artery IJV, internal jugular vein; LCa, longus capitis muscle; LCo, longus coli muscle; LS1, levator scapulae muscle from C1; LS2, Levator scapulae from C2; S, superior cervical ganglion; SAN, spinal accessory nerve; SC, splenius cervicis; SCMc, sternocleidomastoid muscle clavicular head; SCMs, sternocleidomastoid muscle sternal head; SH, stylohyoid muscle; V, vagus nerve; VA, vertebral artery. D. Thirty-gauge needle in position for injection deep to the sternocleidomastoid muscle. Expansion of the space can be seen.

Figure 1 shows the key stages of injection and anatomy. The standard ultrasound image is 1A, the Doppler view is 1B, structures are labeled in 1C, and in 1D, a 30-gauge needle can be seen expanding the space under the investing fascia of the SCM. Consistent with usual cervical plexus block methods, no attempt was made to directly approach the nearby sympathetic trunk with the needle tip [23]

Video 1 depicts the injection process and what it looks like to hydrodissect tissue just deep to the SCM. 

**Video 1:** Ultrasound-guided cervical plexus hydrodissection using a 1-inch needle.

**Figure 2:**
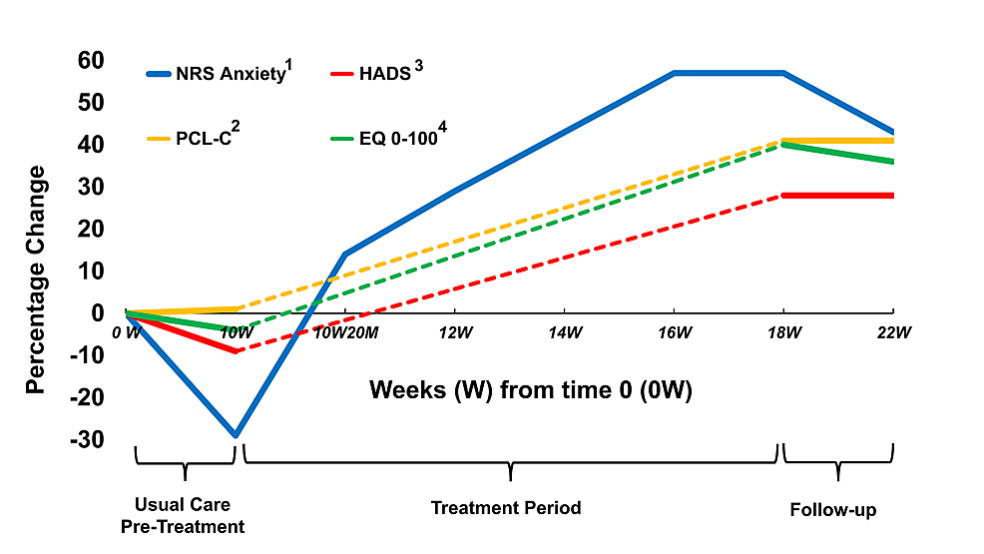
Percentage improvement in NRS Anxiety, HADS, PCL-C, and EuroQol 0-100 scores from baseline through 10 weeks of usual care, followed by biweekly treatment. ^1^ NRS anxiety change from 0-22 weeks was 3 (7 to 4), with an MCID of 1.5 in studies on pain [25], and 1.3 in spasticity [24]. ^2^ PCL-C score change from 0-22 weeks was 30 (73 to 43), with an MCID of 8 in PTSD [26]. ^3^ HADS score change from 0-22 weeks was 7 (32 to 25), with an MCID of 1.5 in COPD [27] and 1.7 in cardiac disease [28]. ^4^ EuroQol 0-100 quality of life scale change from 0-22 weeks was 18; MCID not established. NRS, Numerical Rating Scale; HADS, Hospital Anxiety and Depression Scale; PCL-C, PTSD checklist for civilians

Figure 2 depicts percentage changes from baseline over time in all variables. During the 10-week waiting period, despite an escalation of medication dosages, the patient reported worse 0-10 NRS anxiety (7 to 9 points) and HADS (32 to 35 points), and stable EuroQol 0-100 score (50 to 52 points) and PCL-C score (73 to 72 points). Within 20 minutes of the first injection, the NRS anxiety score improved from 9 to 6. By 18 weeks, NRS anxiety, PCL-C, EuroQol 0-100 score, and HADS scores improved from baseline by 57%, 41%, 40%, and 28%, respectively. These changes met or exceeded twice the minimal clinically important difference (MCID) for the 0-10 NRS [24,25], PCL-C [26], and HADS [27,28], with the MCID for EuroQol 0-100 not yet established. Improvements were stable through 22 weeks.

**Figure 3:**
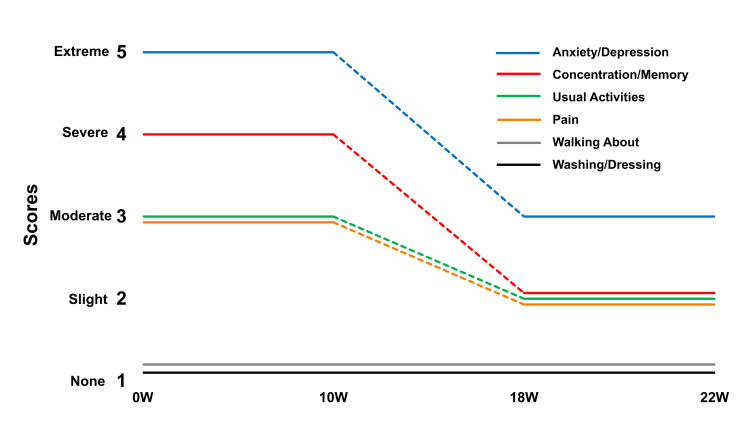
Change in EuroQol 5-dimension, 5-level, plus cognition scores over time. The EuroQol 5-dimension, 5-level, plus cognition scoring, in addition to asking for a number from 0-100 to represent the overall quality of life, asks scorers to indicate a rating from 1-5 in severity for effects on a total of six domains (with cognition added). Only the domain titles as seen here are presented for scoring purposes.

Figure 3, a graph of changes in the EuroQol 5-dimension, 5-level, plus cognition scores shows improvement to a rating of moderate problems only for anxiety-depression, and slight problems with concentration/memory, usual daily activities, and pain.

Four months after his last treatment, the patient was asked to make some retrospective general comments about treatment effects on their life. This is a portion of their unedited comments: "At the first treatment I was nervous, wondering who is this doctor who is going to stick a needle in my neck. I thought I felt different but two weeks later, after the 2nd treatment, I knew I felt much better. Noises did not bother me as much. I found relationships more relaxed as I was not constantly feeling threatened or that something bad was about to happen. My need to self-medicate markedly decreased. Demons visited me much less at night. My mind was not racing or getting spun off as much. While driving I was checking out mentally less often and was not agitated by other drivers' actions. It's been about four months since my last treatment and I am noticing some seeping back in of symptoms, but I'll wait a while for a booster as I have some traveling to do with family for the next month or so."

## Discussion

This patient with PTSD who received DHD-CP reported clinically important improvement in validated outcomes for anxiety, depression, quality of life, and standard PTSD severity measures. Improvements with DHD-CP cannot reasonably be attributed to medication dosage modifications by his psychiatrist 12 weeks prior to the onset of injection treatment, due to the time frame of peak effects of these medications [20-22]. In addition, anxiety improvements were recognized as significant by the patient within 20 minutes of injection, with continued improvement at two-week treatment intervals. This is the first report of dextrose hydrodissection at the typical level for cervical plexus block in order to treat PTSD. It is important to note that anatomical dissection confirms that both left and right superior cervical ganglia are approximately 3 cm long, 0.7 cm wide, and situated in front of the transverse process at C2-3 [29], which is approximated by the angle of the mandible [30, 31]. The ultrasound image in Figure 1 shows the C3 transverse tubercle and the position of the ascending sympathetic truck/superior cervical ganglia just under the deep cervical fascia. Pandit et al. demonstrated that methylene blue dye injected deep to the posterior border of the SCM in the process of a typical cervical plexus block rapidly diffuses across the deep cervical fascia and is found coating scalene muscles, phrenic nerve, and cervical nerve roots [23]. Thus, the injections in this patient would have been expected to expose the superior cervical plexus to D5W. Given the function of the cervical sympathetic chain as a connection/conduit between the central autonomic brain network and the rest of the body, we propose that D5W exposure downregulates sympathetic C fibers responsible for maintenance of a fight or flight response.

A therapeutic effect of D5W on painful/dysfunctional peripheral mixed motor/sensory or sensory nerves, is becoming increasingly apparent in current literature. A primary goal in such treatment is to improve the function of the C-fiber component of such nerves, similar in structure and function to autonomic C-fibers [32]. A direct neurogenic effect of D5W injection on sensory peripheral nerves was first proposed by Dr. John Lyftogt, who injected subcutaneously to treat chronic pain, and reported several consecutive favorable patient data collections [33-35]. Hydrodissection with D5W results in clinical benefit by injection about mixed sensory/motor nerves in the presence of carpal tunnel [36-38] and cubital tunnel syndromes [39] as corroborated by multiple meta-analyses [40-42]. D5W outperforms both saline [37] and steroid [38] as an injectate, pointing toward a direct physiologic benefit of D5W, rather than a mere mechanical effect. The presence of a beneficial physiologic/neurogenic mechanism of D5W is supported by the demonstration of an analgesic response within 20 minutes of caudal epidural injection of D5W versus saline in participants with chronic low back and buttock or leg pain [43], with a consistent analgesic response upon repeat injection, and cumulative clinical benefit [44].

The physiologic mechanism of a beneficial neurogenic effect of D5W is unclear. Multiple interactive physiologic effects of dextrose are more likely than a single mechanism of effect. Some have suggested that dextrose corrects a local glycopenic state affecting energy-demanding overactive C fibers [45]. Dextrose metabolism serves as the primary source of adenosine triphosphate to power the ion pump responsible for maintaining neuronal transmembrane potential [46]. Given the sustained increase in C-fiber firing rates documented in enhanced alertness states such as PTSD or panic disorder [47], coupled with their high baseline energy need [46], a local glycopenic state would not be surprising. MacIver found that local dextrose deficiency results in a marked increase in C-fiber firing rates within 20 minutes, and a return to normal firing rates equally fast with re-provision of dextrose [48]. Our patient reported herein indicated anxiety changes within 20 minutes, consistent with a glycopenic-correction hypothesis of dextrose injection. 

An increasingly common injection approach for the treatment of PTSD by injection is stellate ganglion block (SGB), using an anesthetic agent. A recent narrative review suggested its efficacy [49]. Of interest is that a large case collection by Mulvaney et al. suggested superior efficacy when a portion of the injectate was placed at a second, higher level, targeting the cervical sympathetic chain at the C4 level [50], not far below the single injection level used in this patient. Widespread use of SGB is limited somewhat by the need for a rapid response team in the context of rare but potentially lethal intra-vascular anesthetic injection. One advantage of using dextrose is that DHD-CP can be performed bilaterally, whereas bilateral SGB is impractical due to the unfavorable effects of lidocaine on cardiac vagal modulation and baroreflex sensitivity [51]. Lam et al proposed that D5W may have similar or greater neurogenic benefits than local anesthetic injection without the risk of intravascular injection, making it a potentially perineural injection solution option [10], including future use for PTSD treatment pending further efficacy research.

## Conclusions

In this patient with PTSD, an improvement on validated outcomes for anxiety, depression, quality of life, and standard PTSD severity measures followed DHD-CP. While a case report precludes assignation of causality, the chronic nature of the patient's symptoms, worsening during ten weeks of usual care, and progressive improvement with therapy suggests DHD-CP may have had a role. DCP-HD is quickly learned, appears satisfactory and comfortable to patients, and can be performed bilaterally. This case suggests that more formal assessment of DCP-HD is necessary.
